# Occipital Nerve Stimulation (ONS) for the treatment of chronic headache syndromes

**DOI:** 10.1186/1129-2377-14-S1-P193

**Published:** 2013-02-21

**Authors:** J Vesper, G Bara, TM Kinfe, S Schu

**Affiliations:** 1 Centre of Neuromodulation, Germany; 2 Functional Neurosurgery, Germany

## Introduction

Migraine is highly prevalent along with the high percentage of treatment-refractory cases. ONS may provide pain relief for patients with otherwise refractory primary headache disorders. It is more generally applicable than other invasive methods.

## Objectives

We therefore investigated ONS in a series of patients to determine efficacy, complications and outcome.

## Methods

We included a case series of 20 patients who had chronic headaches for a duration of 5.3 y who underwent ONS lead implantation (SJM, Octrode). Prior to surgery patients had received conservative and surgical therapies including antidepressants, occipital nerve blocks, opioids, cervical posterior fusion (one patient), without success. 9 patients suffered from chronic migraine, 1 had a history of thalamic infarction, 1 patient suffered from cluster headache, 4 patients complained tension headache and 5 patients with recurrent cervicozephalgia after spine surgery. Using a midline approach two octrodes were placed subcutaneously and positioned across the level of C1 using fluoroscopy. Leads were placed under general anesthesia and externalized for three days.

## Results

Device dislocation was found in 3 cases. 16 patients mentioned significant relief of pain, so that they all underwent insertion of the generator (eon MINI, SJM), in 3 patients 30% pain reduction was achieved, one patient did not benefit. Decreases in pain led to an improvement in functional capacity during the 3 months follow-up after implantation. The mean VAS score changed from 8.2 ± 1.5 to 3.5 ± 1.3 at the 6 months follow-up. No complicaoccurred.

## Discussion

The exact mechanism of neuromodulation in the tratment of different headache syndromes remains unclear. ONS is safe and efficacious in the treatment of medically intractable headaches conditions. Further investigations are required to evaluate predictor for patient selection and sstimulation setting among this crucial pain conditions.

## Conflict of interest

SS and JV received consultant fees, GB received a fellowship stipendium from St. Jude Medical.
